# Supplementary material for: Overlapping cell population expression profiling and regulatory inference in C. elegans
Source: BMC Genomics. 2016 Feb 29;17:159. doi: 10.1186/s12864-016-2482-z (PMC4772325; doi:10.1186/s12864-016-2482-z)
Supplement: Additional file 13: — Web supplement. (DOC 21 kb) [file 12864_2016_2482_MOESM13_ESM.zip › sortWeb/clusters/hier.300.clusters/299.html]

Cluster 299 

## Cluster 299

### Expression

| cnd-1 rep. 1 | cnd-1 rep. 2 | cnd-1 rep. 3 | pha-4 rep. 1 | pha-4 rep. 2 | pha-4 rep. 3 | ceh-27 | ceh-36 | ceh-6 | F21D5.9 | mir-57 | mls-2 | pal-1 | pros-1 | ttx-3 | unc-130 | hlh-16 | irx-1 | ceh-6 (+) hlh-16 (+) | ceh-6 (+) hlh-16 (-) | ceh-6 (-) hlh-16 (+) | cnd-1 singlets | pha-4 singlets | 0 | 60 | 120 | 150 | 180 | 240 | 330 | 390 | 420 | 480 | 540 | 570 | 600 | 630 | 660 | NAME | Functional description |
| --- | --- | --- | --- | --- | --- | --- | --- | --- | --- | --- | --- | --- | --- | --- | --- | --- | --- | --- | --- | --- | --- | --- | --- | --- | --- | --- | --- | --- | --- | --- | --- | --- | --- | --- | --- | --- | --- | --- | --- |
|  |  |  |  |  |  |  |  |  |  |  |  |  |  |  |  |  |  |  |  |  |  |  |  |  |  |  |  |  |  |  |  |  |  |  |  |  |  | Y6E2A.4 |  |
|  |  |  |  |  |  |  |  |  |  |  |  |  |  |  |  |  |  |  |  |  |  |  |  |  |  |  |  |  |  |  |  |  |  |  |  |  |  | K09C4.9 |  |
|  |  |  |  |  |  |  |  |  |  |  |  |  |  |  |  |  |  |  |  |  |  |  |  |  |  |  |  |  |  |  |  |  |  |  |  |  |  | Y60C6A.1 |  |
|  |  |  |  |  |  |  |  |  |  |  |  |  |  |  |  |  |  |  |  |  |  |  |  |  |  |  |  |  |  |  |  |  |  |  |  |  |  | *irld-60* | Insulin/EGF-Receptor L Domain protein |
|  |  |  |  |  |  |  |  |  |  |  |  |  |  |  |  |  |  |  |  |  |  |  |  |  |  |  |  |  |  |  |  |  |  |  |  |  |  | Y57G11C.44 |  |
|  |  |  |  |  |  |  |  |  |  |  |  |  |  |  |  |  |  |  |  |  |  |  |  |  |  |  |  |  |  |  |  |  |  |  |  |  |  | C08E8.6 |  |
|  |  |  |  |  |  |  |  |  |  |  |  |  |  |  |  |  |  |  |  |  |  |  |  |  |  |  |  |  |  |  |  |  |  |  |  |  |  | F25H8.8 |  |
|  |  |  |  |  |  |  |  |  |  |  |  |  |  |  |  |  |  |  |  |  |  |  |  |  |  |  |  |  |  |  |  |  |  |  |  |  |  | *sqst-3* | SeQueSTosome related |
|  |  |  |  |  |  |  |  |  |  |  |  |  |  |  |  |  |  |  |  |  |  |  |  |  |  |  |  |  |  |  |  |  |  |  |  |  |  | *hex-1* | HEXosaminidase |
|  |  |  |  |  |  |  |  |  |  |  |  |  |  |  |  |  |  |  |  |  |  |  |  |  |  |  |  |  |  |  |  |  |  |  |  |  |  | E04D5.2 |  |
|  |  |  |  |  |  |  |  |  |  |  |  |  |  |  |  |  |  |  |  |  |  |  |  |  |  |  |  |  |  |  |  |  |  |  |  |  |  | C41G11.1 |  |
|  |  |  |  |  |  |  |  |  |  |  |  |  |  |  |  |  |  |  |  |  |  |  |  |  |  |  |  |  |  |  |  |  |  |  |  |  |  | C13C4.6 |  |
|  |  |  |  |  |  |  |  |  |  |  |  |  |  |  |  |  |  |  |  |  |  |  |  |  |  |  |  |  |  |  |  |  |  |  |  |  |  | B0393.9 |  |
|  |  |  |  |  |  |  |  |  |  |  |  |  |  |  |  |  |  |  |  |  |  |  |  |  |  |  |  |  |  |  |  |  |  |  |  |  |  | *mec-3* | MEChanosensory abnormality |
|  |  |  |  |  |  |  |  |  |  |  |  |  |  |  |  |  |  |  |  |  |  |  |  |  |  |  |  |  |  |  |  |  |  |  |  |  |  | M03E7.1 |  |
|  |  |  |  |  |  |  |  |  |  |  |  |  |  |  |  |  |  |  |  |  |  |  |  |  |  |  |  |  |  |  |  |  |  |  |  |  |  | F33C8.4 |  |
|  |  |  |  |  |  |  |  |  |  |  |  |  |  |  |  |  |  |  |  |  |  |  |  |  |  |  |  |  |  |  |  |  |  |  |  |  |  | C33D9.10 |  |
|  |  |  |  |  |  |  |  |  |  |  |  |  |  |  |  |  |  |  |  |  |  |  |  |  |  |  |  |  |  |  |  |  |  |  |  |  |  | *twk-42* | TWiK family of potassium channels |
|  |  |  |  |  |  |  |  |  |  |  |  |  |  |  |  |  |  |  |  |  |  |  |  |  |  |  |  |  |  |  |  |  |  |  |  |  |  | B0403.6 |  |
|  |  |  |  |  |  |  |  |  |  |  |  |  |  |  |  |  |  |  |  |  |  |  |  |  |  |  |  |  |  |  |  |  |  |  |  |  |  | F09D12.2 |  |
|  |  |  |  |  |  |  |  |  |  |  |  |  |  |  |  |  |  |  |  |  |  |  |  |  |  |  |  |  |  |  |  |  |  |  |  |  |  | *nhr-6* | Nuclear Hormone Receptor family |
|  |  |  |  |  |  |  |  |  |  |  |  |  |  |  |  |  |  |  |  |  |  |  |  |  |  |  |  |  |  |  |  |  |  |  |  |  |  | *des-2* | DEgeneration Suppressor |
|  |  |  |  |  |  |  |  |  |  |  |  |  |  |  |  |  |  |  |  |  |  |  |  |  |  |  |  |  |  |  |  |  |  |  |  |  |  | *deg-3* | DEGeneration of certain neurons |
|  |  |  |  |  |  |  |  |  |  |  |  |  |  |  |  |  |  |  |  |  |  |  |  |  |  |  |  |  |  |  |  |  |  |  |  |  |  | ZK1010.5 |  |
|  |  |  |  |  |  |  |  |  |  |  |  |  |  |  |  |  |  |  |  |  |  |  |  |  |  |  |  |  |  |  |  |  |  |  |  |  |  | C55B6.4 |  |
|  |  |  |  |  |  |  |  |  |  |  |  |  |  |  |  |  |  |  |  |  |  |  |  |  |  |  |  |  |  |  |  |  |  |  |  |  |  | *glt-1* | GLutamate Transporter family |
|  |  |  |  |  |  |  |  |  |  |  |  |  |  |  |  |  |  |  |  |  |  |  |  |  |  |  |  |  |  |  |  |  |  |  |  |  |  | C06C3.3 |  |
|  |  |  |  |  |  |  |  |  |  |  |  |  |  |  |  |  |  |  |  |  |  |  |  |  |  |  |  |  |  |  |  |  |  |  |  |  |  | F58H7.1 |  |
|  |  |  |  |  |  |  |  |  |  |  |  |  |  |  |  |  |  |  |  |  |  |  |  |  |  |  |  |  |  |  |  |  |  |  |  |  |  | F44E2.10 |  |
|  |  |  |  |  |  |  |  |  |  |  |  |  |  |  |  |  |  |  |  |  |  |  |  |  |  |  |  |  |  |  |  |  |  |  |  |  |  | F53F8.7 |  |
|  |  |  |  |  |  |  |  |  |  |  |  |  |  |  |  |  |  |  |  |  |  |  |  |  |  |  |  |  |  |  |  |  |  |  |  |  |  | *bas-1* | Biogenic Amine Synthesis related |
|  |  |  |  |  |  |  |  |  |  |  |  |  |  |  |  |  |  |  |  |  |  |  |  |  |  |  |  |  |  |  |  |  |  |  |  |  |  | *tkr-3* | TachyKinin Receptor family |
|  |  |  |  |  |  |  |  |  |  |  |  |  |  |  |  |  |  |  |  |  |  |  |  |  |  |  |  |  |  |  |  |  |  |  |  |  |  | Y47G6A.13 |  |
|  |  |  |  |  |  |  |  |  |  |  |  |  |  |  |  |  |  |  |  |  |  |  |  |  |  |  |  |  |  |  |  |  |  |  |  |  |  | *sma-10* | SMAll |
|  |  |  |  |  |  |  |  |  |  |  |  |  |  |  |  |  |  |  |  |  |  |  |  |  |  |  |  |  |  |  |  |  |  |  |  |  |  | *flp-21* | FMRF-Like Peptide |
|  |  |  |  |  |  |  |  |  |  |  |  |  |  |  |  |  |  |  |  |  |  |  |  |  |  |  |  |  |  |  |  |  |  |  |  |  |  | T22B7.7 |  |
|  |  |  |  |  |  |  |  |  |  |  |  |  |  |  |  |  |  |  |  |  |  |  |  |  |  |  |  |  |  |  |  |  |  |  |  |  |  | F36D4.4 |  |
|  |  |  |  |  |  |  |  |  |  |  |  |  |  |  |  |  |  |  |  |  |  |  |  |  |  |  |  |  |  |  |  |  |  |  |  |  |  | C54G7.1 |  |
|  |  |  |  |  |  |  |  |  |  |  |  |  |  |  |  |  |  |  |  |  |  |  |  |  |  |  |  |  |  |  |  |  |  |  |  |  |  | *srr-3* | Serpentine Receptor, class R |
|  |  |  |  |  |  |  |  |  |  |  |  |  |  |  |  |  |  |  |  |  |  |  |  |  |  |  |  |  |  |  |  |  |  |  |  |  |  | C56C10.9 |  |
|  |  |  |  |  |  |  |  |  |  |  |  |  |  |  |  |  |  |  |  |  |  |  |  |  |  |  |  |  |  |  |  |  |  |  |  |  |  | F53B7.7 |  |
|  |  |  |  |  |  |  |  |  |  |  |  |  |  |  |  |  |  |  |  |  |  |  |  |  |  |  |  |  |  |  |  |  |  |  |  |  |  | *tbh-1* | Tyramine Beta Hydroxylase |
|  |  |  |  |  |  |  |  |  |  |  |  |  |  |  |  |  |  |  |  |  |  |  |  |  |  |  |  |  |  |  |  |  |  |  |  |  |  | *tdc-1* | Tyrosine DeCarboxylase |
|  |  |  |  |  |  |  |  |  |  |  |  |  |  |  |  |  |  |  |  |  |  |  |  |  |  |  |  |  |  |  |  |  |  |  |  |  |  | T03G11.10 |  |
|  |  |  |  |  |  |  |  |  |  |  |  |  |  |  |  |  |  |  |  |  |  |  |  |  |  |  |  |  |  |  |  |  |  |  |  |  |  | F25D1.2 |  |
|  |  |  |  |  |  |  |  |  |  |  |  |  |  |  |  |  |  |  |  |  |  |  |  |  |  |  |  |  |  |  |  |  |  |  |  |  |  | *ugt-56* | UDP-GlucuronosylTransferase |
|  |  |  |  |  |  |  |  |  |  |  |  |  |  |  |  |  |  |  |  |  |  |  |  |  |  |  |  |  |  |  |  |  |  |  |  |  |  | C30F2.2 |  |
|  |  |  |  |  |  |  |  |  |  |  |  |  |  |  |  |  |  |  |  |  |  |  |  |  |  |  |  |  |  |  |  |  |  |  |  |  |  | T01G6.1 |  |
|  |  |  |  |  |  |  |  |  |  |  |  |  |  |  |  |  |  |  |  |  |  |  |  |  |  |  |  |  |  |  |  |  |  |  |  |  |  | *gpa-11* | G Protein, Alpha subunit |
|  |  |  |  |  |  |  |  |  |  |  |  |  |  |  |  |  |  |  |  |  |  |  |  |  |  |  |  |  |  |  |  |  |  |  |  |  |  | *acc-2* | Acetylcholine-gated Chloride Channel |
|  |  |  |  |  |  |  |  |  |  |  |  |  |  |  |  |  |  |  |  |  |  |  |  |  |  |  |  |  |  |  |  |  |  |  |  |  |  | *cat-2* | abnormal CATecholamine distribution |
|  |  |  |  |  |  |  |  |  |  |  |  |  |  |  |  |  |  |  |  |  |  |  |  |  |  |  |  |  |  |  |  |  |  |  |  |  |  | *dat-1* | DopAmine Transporter |
|  |  |  |  |  |  |  |  |  |  |  |  |  |  |  |  |  |  |  |  |  |  |  |  |  |  |  |  |  |  |  |  |  |  |  |  |  |  | *flp-25* | FMRF-Like Peptide |
|  |  |  |  |  |  |  |  |  |  |  |  |  |  |  |  |  |  |  |  |  |  |  |  |  |  |  |  |  |  |  |  |  |  |  |  |  |  | F59F3.6 |  |
|  |  |  |  |  |  |  |  |  |  |  |  |  |  |  |  |  |  |  |  |  |  |  |  |  |  |  |  |  |  |  |  |  |  |  |  |  |  | *ins-22* | INSulin related |
|  |  |  |  |  |  |  |  |  |  |  |  |  |  |  |  |  |  |  |  |  |  |  |  |  |  |  |  |  |  |  |  |  |  |  |  |  |  | M03D4.3 |  |
|  |  |  |  |  |  |  |  |  |  |  |  |  |  |  |  |  |  |  |  |  |  |  |  |  |  |  |  |  |  |  |  |  |  |  |  |  |  | F09E10.7 |  |
|  |  |  |  |  |  |  |  |  |  |  |  |  |  |  |  |  |  |  |  |  |  |  |  |  |  |  |  |  |  |  |  |  |  |  |  |  |  | *galt-1* | GALacTosyltransferase |
|  |  |  |  |  |  |  |  |  |  |  |  |  |  |  |  |  |  |  |  |  |  |  |  |  |  |  |  |  |  |  |  |  |  |  |  |  |  | *str-180* | Seven TM Receptor |
|  |  |  |  |  |  |  |  |  |  |  |  |  |  |  |  |  |  |  |  |  |  |  |  |  |  |  |  |  |  |  |  |  |  |  |  |  |  | F32B5.6 |  |
|  |  |  |  |  |  |  |  |  |  |  |  |  |  |  |  |  |  |  |  |  |  |  |  |  |  |  |  |  |  |  |  |  |  |  |  |  |  | F14B4.2 |  |
|  |  |  |  |  |  |  |  |  |  |  |  |  |  |  |  |  |  |  |  |  |  |  |  |  |  |  |  |  |  |  |  |  |  |  |  |  |  | *gly-2* | GLYcosylation related |
|  |  |  |  |  |  |  |  |  |  |  |  |  |  |  |  |  |  |  |  |  |  |  |  |  |  |  |  |  |  |  |  |  |  |  |  |  |  | F36G9.4 |  |
|  |  |  |  |  |  |  |  |  |  |  |  |  |  |  |  |  |  |  |  |  |  |  |  |  |  |  |  |  |  |  |  |  |  |  |  |  |  | C18C4.5 |  |
|  |  |  |  |  |  |  |  |  |  |  |  |  |  |  |  |  |  |  |  |  |  |  |  |  |  |  |  |  |  |  |  |  |  |  |  |  |  | T07D1.2 |  |

### Phenotypes enriched

none found

### Anatomy terms enriched

|  |  |  |  |
| --- | --- | --- | --- |
| **Group name** | **Number in cluster** | **Enrichment** | **FDR corrected p** |
| sensory neuron | 13 | 6.58 | 0.000189 |
| posterior lateral right ganglion | 6 | 24.13 | 0.000476 |
| head ganglion | 12 | 5.36 | 0.004270 |
| posterior lateral left ganglion | 6 | 15.08 | 0.006180 |
| posterior lateral ganglion | 6 | 14.92 | 0.006450 |
| embryonic cell | 11 | 4.70 | 0.032300 |

### GO terms enriched

|  |  |  |
| --- | --- | --- |
| **GO term** | **Number of genes** | **FDR-corrected p-value** |
| organic hydroxy compound biosynthetic process | 3 | 0.019 |
| regulation of neurotransmitter levels | 3 | 0.026 |

### Expression clusters enriched

|  |  |  |  |
| --- | --- | --- | --- |
| **Group name** | **Number in cluster** | **Enrichment** | **FDR corrected p** |
| Genes significantly enriched (> 2x, FDR < 5%) in a particular cell-type versus a reference sample of all cells at the same stage. WBPaper00037950:all-neurons\_larva\_enriched | 23 | 5.48 | 4.35e-09 |
| Genes that show selective expression in a subset of cell types vs broadly expressed in many cell types. Correspond to 20% - 57% of enriched\_genes for a given cell type. WBPaper00037950:all-neurons\_larva\_SelectivelyEnriched | 15 | 6.63 | 2.60e-06 |
| Larval Pan-neural Enriched Genes. | 23 | 3.59 | 1.17e-05 |
| Genes significantly enriched (> 2x, FDR < 5%) in a particular cell-type versus a reference sample of all cells at the same stage. WBPaper00037950:coelomocytes\_embryo\_enriched | 13 | 5.49 | 2.03e-04 |
| Genes that show selective expression in a subset of cell types vs broadly expressed in many cell types. Correspond to 20% - 57% of enriched\_genes for a given cell type. WBPaper00037950:coelomocytes\_embryo\_SelectivelyEnriched | 8 | 7.88 | 2.34e-03 |
| Genes significantly enriched (> 2x, FDR < 5%) in a particular cell-type versus a reference sample of all cells at both embryonic and larval stages. WBPaper00037950:dopaminergic-neurons\_CoreEnriched | 6 | 9.65 | 9.77e-03 |
| TGF-beta adult-specific down/IIS up | 5 | 11.94 | 1.54e-02 |
| WT-Pico Pan-neural Enriched Genes, with genes found multiple times in a single dataset removed (without dups). | 18 | 2.71 | 1.56e-02 |
| Genes that show selective expression in a subset of cell types vs broadly expressed in many cell types. Correspond to 20% - 57% of enriched\_genes for a given cell type. WBPaper00037950:dopaminergic-neurons\_embryo\_SelectivelyEnriched | 6 | 8.42 | 1.90e-02 |
| Genes that show selective expression in a subset of cell types vs broadly expressed in many cell types. Correspond to 20% - 57% of enriched\_genes for a given cell type. WBPaper00037950:dopaminergic-neurons\_larva\_SelectivelyEnriched | 9 | 4.72 | 2.62e-02 |

### Motifs enriched

|  |  |  |  |  |  |
| --- | --- | --- | --- | --- | --- |
| **Motif** | **Logo** | **Possible orthologs** | **Number of motifs in cluster** | **Enrichment** | **FDR corrected p** |
| Sox15\_3457 |  | sox-4 | 38 | 1.87 | 0.0010 |
| V$NKX25\_02 |  | ceh-8 ceh-24 ceh-1 ceh-9 ceh-31 lim-7 | 26 | 2.39 | 0.0013 |
| Sry\_2833 |  | sox-4 pop-1 gei-3 C05C9.3 | 22 | 2.70 | 0.0013 |
| Etv4 |  | lin-1 C24A1.2 | 20 | 2.88 | 0.0015 |
| FOXJ2\_1 |  | let-381 (0.77) lin-31 (0.57) daf-16 | 38 | 1.84 | 0.0015 |
| Erg |  | lin-1 C24A1.2 | 21 | 2.70 | 0.0020 |
| Hmx1\_3423 |  | ceh-9 | 10 | 5.22 | 0.0029 |
| ELK4\_f1 |  | lin-1 | 19 | 2.83 | 0.0029 |
| pTH9246 |  | C34D1.1 | 18 | 2.90 | 0.0036 |
| FOXB1\_2 |  | lin-31 (0.57) ceh-43 | 27 | 2.17 | 0.0038 |
| pTH9189 |  | dmd-3 | 45 | 1.58 | 0.0039 |
| ftz\_FlyReg\_FBgn0001077 |  | alr-1 (0.56) ceh-43 ceh-1 lin-39 ceh-31 | 16 | 3.11 | 0.0045 |
| MA0039.2 |  | klf-1 klf-2 | 35 | 1.80 | 0.0057 |
| pTH9261 |  | dmd-3 | 37 | 1.74 | 0.0065 |
| Pax7\_3783 |  | alr-1 (0.56) | 26 | 2.14 | 0.0066 |
| pTH9198 |  | dmd-3 | 39 | 1.68 | 0.0067 |
| pTH9247 |  | dmd-3 C34D1.1 | 36 | 1.75 | 0.0071 |
| Dlx3\_1030 |  | ceh-43 | 9 | 4.94 | 0.0089 |
| Foxj1\_3125 |  | let-381 (0.77) lin-31 (0.57) fkh-10 | 46 | 1.51 | 0.0090 |
| V$RSRFC4\_01 |  | mef-2 | 23 | 2.25 | 0.0092 |
| PAX5\_1 |  | pax-2 | 33 | 1.81 | 0.0095 |
| Hlxb9\_3422 |  | ceh-12 | 9 | 4.88 | 0.0095 |
| pTH9215 |  | C34D1.1 | 43 | 1.56 | 0.0100 |
| Bsx\_3483 |  | ceh-31 | 19 | 2.52 | 0.0100 |
| Dlx5\_3419 |  | alr-1 (0.56) ceh-43 ceh-12 ceh-45 | 15 | 2.97 | 0.0110 |
| SOX2\_5 |  | sox-4 | 54 | 1.36 | 0.0110 |
| V$FOXJ2\_01 |  | lin-31 (0.57) | 33 | 1.79 | 0.0110 |
| CG4328\_Cell\_FBgn0036274 |  | alr-1 (0.56) ceh-14 ceh-43 ceh-1 ceh-16 lin-39 ceh-31 ceh-12 lim-6 lim-7 | 24 | 2.15 | 0.0120 |
| I$UBX\_01 |  | lin-39 | 21 | 2.33 | 0.0120 |
| pTH10816 |  | dmd-6 | 32 | 1.81 | 0.0120 |
| Msx3\_3206 |  | ceh-1 lin-39 eyg-1 | 15 | 2.93 | 0.0120 |
| NR1D1\_f1 |  | nhr-213 nhr-118 | 35 | 1.72 | 0.0130 |
| PROX1\_1 |  | ceh-26 | 9 | 4.64 | 0.0130 |
| pTH9250 |  | C34D1.1 | 37 | 1.67 | 0.0130 |
| pTH5919 |  | irx-1 | 26 | 2.03 | 0.0130 |
| HXA10\_f1 |  | lin-39 | 28 | 1.94 | 0.0130 |
| pTH8330 |  | C34D1.1 | 23 | 2.18 | 0.0130 |
| V$AP4\_Q5 |  | hlh-11 | 12 | 3.48 | 0.0140 |
| Elf4 |  | C24A1.2 | 19 | 2.44 | 0.0150 |
| POU3F3\_1 |  | ceh-18 (0.51) | 31 | 1.82 | 0.0150 |
| NR2F6\_f1 |  | nhr-2 | 57 | 1.30 | 0.0150 |
| Nkx1-1\_3856 |  | ceh-30 | 14 | 2.99 | 0.0170 |
| RFX2\_1 |  | daf-19 | 24 | 2.08 | 0.0170 |
| Hoxa7\_3750 |  | lin-39 | 25 | 2.03 | 0.0170 |
| V$XFD3\_01 |  | let-381 (0.77) | 11 | 3.60 | 0.0180 |
| klu\_SANGER\_10\_FBgn0013469 |  | ZC328.2 (0.73) | 16 | 2.68 | 0.0180 |
| pTH10797 |  | K11D2.4 | 32 | 1.76 | 0.0200 |
| NR4A2\_3 |  | nhr-6 (0.82) nhr-71 nhr-68 | 33 | 1.73 | 0.0200 |
| FOXO1\_si |  | irx-1 daf-16 | 25 | 2.00 | 0.0210 |
| MA0204.1 |  | dmd-4 ceh-32 | 28 | 1.88 | 0.0210 |
| pTH5976 |  | irx-1 | 17 | 2.51 | 0.0220 |
| Irx3\_2226 |  | irx-1 | 13 | 3.05 | 0.0220 |
| Barx2\_3447 |  | ceh-43 | 23 | 2.09 | 0.0220 |
| Irx3\_0920 |  | irx-1 | 16 | 2.60 | 0.0230 |
| pTH10777 |  | dmd-3 | 35 | 1.66 | 0.0240 |
| Nkx2-6\_3437 |  | ceh-24 dsc-1 | 26 | 1.93 | 0.0240 |
| Hoxc8\_3429 |  | lin-39 | 25 | 1.97 | 0.0250 |
| Etv3 |  | lin-1 | 18 | 2.37 | 0.0270 |
| HXD10\_f1 |  | php-3 | 19 | 2.29 | 0.0270 |
| Mw160 |  | nhr-68 | 35 | 1.64 | 0.0280 |
| Barx1\_2877 |  | ceh-43 | 7 | 5.25 | 0.0280 |
| Sp4\_1011 |  | sptf-3 klf-2 | 24 | 1.99 | 0.0280 |
| MA0161.1 |  | nfi-1 | 27 | 1.87 | 0.0290 |
| pTH10722 |  | egrh-3 | 29 | 1.80 | 0.0300 |
| BARHL2\_6 |  | ceh-31 | 25 | 1.94 | 0.0300 |
| V$S8\_01 |  | ceh-45 | 24 | 1.97 | 0.0320 |
| Dlx2\_2273 |  | ceh-43 | 11 | 3.29 | 0.0320 |
| pTH10837 |  | T22H9.4 | 29 | 1.78 | 0.0340 |
| pTH1739 |  | nhr-255 | 15 | 2.59 | 0.0340 |
| pTH5064 |  | aha-1 | 33 | 1.67 | 0.0350 |
| Nr2f6\_1 |  | nhr-2 nhr-62 | 23 | 2.00 | 0.0350 |
| pTH9237 |  | mel-28 | 49 | 1.37 | 0.0370 |
| pTH10647 |  | nhr-232 | 37 | 1.57 | 0.0370 |
| MA0222.1 |  | ceh-20 ceh-32 | 16 | 2.46 | 0.0370 |
| V$TATA\_01 |  | tbp-1 | 38 | 1.55 | 0.0380 |
| Foxj3\_1 |  | lin-31 (0.57) | 37 | 1.57 | 0.0390 |
| HXA9\_f1 |  | lin-39 ceh-32 | 28 | 1.79 | 0.0390 |
| pTH2193 |  | nhr-2 | 11 | 3.19 | 0.0390 |
| IRX2\_1 |  | irx-1 | 27 | 1.82 | 0.0410 |
| NR4A2\_1 |  | nhr-6 (0.82) | 15 | 2.53 | 0.0420 |
| SOX10\_1 |  | sox-4 | 42 | 1.47 | 0.0420 |
| pTH6482 |  | ceh-19 | 11 | 3.13 | 0.0430 |
| V$VMYB\_01 |  | D1081.8 | 10 | 3.38 | 0.0440 |
| Hoxb6\_3428 |  | lin-39 | 21 | 2.05 | 0.0440 |
| Sox4 |  | pop-1 | 38 | 1.53 | 0.0440 |
| V$GATA3\_02 |  | elt-1 | 41 | 1.48 | 0.0460 |
| pTH6569 |  | ceh-43 | 23 | 1.95 | 0.0460 |
| Hoxc10\_2 |  | php-3 | 45 | 1.41 | 0.0470 |
| pTH10811 |  | nhr-216 (0.58) | 25 | 1.86 | 0.0470 |
| Irx5\_2385 |  | irx-1 | 30 | 1.70 | 0.0470 |
| pTH3469 |  | nhr-213 | 31 | 1.68 | 0.0470 |
| Eip74EF\_FlyReg\_FBgn0000567 |  | C24A1.2 | 33 | 1.63 | 0.0480 |
| En1\_3123 |  | ceh-16 | 10 | 3.30 | 0.0500 |

### Correlated (and anti-correlated) transcription factors

|  |  |
| --- | --- |
| **Transcription factor** | **Correlation** |
| nhr-6 | 0.82 |
| ceh-90 | 0.78 |
| mec-3 | 0.77 |
| let-381 | 0.77 |
| nhr-187 | 0.75 |
| che-1 | 0.75 |
| ZC328.2 | 0.73 |
| nhr-192 | 0.70 |
| hlh-4 | 0.70 |
| nhr-188 | 0.70 |
| Y22D7AL.16 | 0.67 |
| npax-2 | 0.63 |
| ceh-33 | 0.62 |
| nhr-166 | 0.62 |
| hlh-33 | 0.62 |
| moe-3 | 0.61 |
| F13H6.1 | 0.60 |
| ceh-49 | 0.59 |
| C02F12.5 | 0.58 |
| nhr-216 | 0.58 |
| ahr-1 | 0.57 |
| lin-31 | 0.57 |
| dhhc-2 | 0.57 |
| alr-1 | 0.56 |
| lfi-1 | 0.56 |
| ZK185.1 | -0.40 |
| pqn-75 | -0.41 |
| lin-40 | -0.41 |
| Y95B8A.7 | -0.41 |
| nhr-80 | -0.42 |
| ceh-51 | -0.42 |
| F49E8.2 | -0.42 |
| ZK1067.2 | -0.43 |
| nhr-150 | -0.44 |
| cey-3 | -0.44 |
| pax-3 | -0.45 |
| tbx-31 | -0.45 |
| ztf-4 | -0.45 |
| cebp-2 | -0.46 |
| nhr-171 | -0.46 |
| R10E4.11 | -0.47 |
| ztf-28 | -0.51 |
| nhr-246 | -0.51 |
| cep-1 | -0.54 |
| cey-2 | -0.55 |
| D2030.7 | -0.55 |
| hmg-12 | -0.56 |
| ccch-3 | -0.58 |
| Y57A10A.31 | -0.58 |
| ubxn-1 | -0.59 |

### ChIP peaks enriched

none found
